# The BCGΔBCG1419c Vaccine Candidate Reduces Lung Pathology, IL-6, TNF-α, and IL-10 During Chronic TB Infection

**DOI:** 10.3389/fmicb.2018.01281

**Published:** 2018-06-12

**Authors:** Mario A. Flores-Valdez, César Pedroza-Roldán, Michel de Jesús Aceves-Sánchez, Eliza J. R. Peterson, Nitin S. Baliga, Rogelio Hernández-Pando, JoLynn Troudt, Elizabeth Creissen, Linda Izzo, Helle Bielefeldt-Ohmann, Thomas Bickett, Angelo A. Izzo

**Affiliations:** ^1^Biotecnología Médica y Farmacéutica, Centro de Investigación y Asistencia en Tecnología y Diseño del Estado de Jalisco, Guadalajara, Mexico; ^2^Departamento de Medicina Veterinaria, Centro Universitario de Ciencias Biológicas y Agropecuarias, Universidad de Guadalajara, Zapopan, Mexico; ^3^Institute for Systems Biology, Seattle, WA, United States; ^4^Sección de Patología Experimental, Instituto Nacional de Ciencias Médicas y Nutrición Salvador Zubirán, Mexico City, Mexico; ^5^Department of Microbiology, Immunology and Pathology, Colorado State University, Fort Collins, CO, United States; ^6^Australian Infectious Diseases Research Centre, The University of Queensland, Saint Lucia, QLD, Australia; ^7^School of Veterinary Science, The University of Queensland, Brisbane, QLD, Australia

**Keywords:** tuberculosis, latent TB infection, BCG, pellicles, biofilms, c-di-GMP

## Abstract

*Mycobacterium tuberculosis* (*M. tuberculosis*), the causative agent of human tuberculosis (TB), is estimated to be harbored by up to 2 billion people in a latent TB infection (LTBI) state. The only TB vaccine approved for use in humans, BCG, does not confer protection against establishment of or reactivation from LTBI, so new vaccine candidates are needed to specifically address this need. Following the hypothesis that mycobacterial biofilms resemble aspects of LTBI, we modified BCG by deleting the *BCG1419c* gene to create the BCGΔBCG1419c vaccine strain. In this study, we compared cytokine profiles, bacterial burden, and lung lesions after immunization with BCG or BCGΔBCG1419c before and after 6 months of aerosol infection with *M. tuberculosis* H37Rv in the resistant C57BL/6 mouse model. Our results show that in infected mice, BCGΔBCG1419c significantly reduced lung lesions and IL-6 in comparison to the unmodified BCG strain, and was the only vaccine that decreased production of TNF-α and IL-10 compared to non-vaccinated mice, while vaccination with BCG or BCGΔBCG1419c significantly reduced IFN-γ production. Moreover, transcriptome profiling of BCGΔBCG1419c suggests that compared to BCG, it has decreased expression of genes involved in mycolic acids (MAs) metabolism, and antigenic chaperones, which might be involved in reduced pathology compared to BCG-vaccinated mice.

## Introduction

*Mycobacterium tuberculosis* (*M. tuberculosis*), the etiological agent of human tuberculosis (TB), remains a major challenge to public health. According to the World Health Organization, there were 10.4 million new TB cases around the world in 2015, and an estimated 1.4 million associated deaths (WHO, 2016). Currently, the only TB vaccine approved for use in humans is the *Mycobacterium bovis*-derived Bacillus Calmette–Guerin (BCG), which elicits variable levels of protection (Smith et al., 2016) and lacks efficacy versus pulmonary and latent TB infection (LTBI), therefore, several efforts have been made to develop new TB vaccine candidates (Kaufmann et al., 2017a), including preventive pre-exposure vaccines, preventive post-exposure vaccines, and therapeutic vaccines which can be administered along with TB drugs (Kaufmann et al., 2017a). BCG induces high level of T-cell immunity after administration, but only modest levels of central memory T cells in lungs (Kaufmann et al., 2017a). Hence, new approaches aim to increase the production of long-term memory T cells by using antigens as subunit vaccines (Kaufmann et al., 2017a).

Focusing on live attenuated vaccine candidates that have been evaluated for efficacy versus chronic infection, as is the intended primary use of our BCGΔBCG1419c vaccine candidate, pre-infection administration of a rBCG expressing the membrane-perforating listeriolysin (rBCG *hly*^+^) derived from *L. monocytogenes*, resulted in a higher than 1-log_10_ drop in bacilli CFUs compared to non-vaccinated BALB/c mice (Grode et al., 2005), and post-exposure administration of the same rBCG promoted a 1-log_10_ reduction of bacillary burden in lungs, compared to non-vaccinated mice, and 0.5-log_10_ reduction compared to parental BCG, after 14 weeks of deprivation of antibiotic treatment in a subclinical TB infection model (Gengenbacher et al., 2016). Of note, the effect in protection versus lung pathology conferred upon vaccination and post-infection was not assessed.

In order to explore the hypothesis that mycobacterial biofilms resemble aspects of chronic TB infection (Flores-Valdez, 2016), we deleted the cyclic di-GMP phosphodiesterase-encoding gene *BCG1419c*, to create the BCGΔBCG1419c Pasteur-derivative strain (Flores-Valdez et al., 2015). We previously showed that in immunocompromised nu/nu mice, BCGΔBCG1419c was as safe as parental BCG, and that in a mouse model of progressive infection with *M. tuberculosis* H37Rv, compared to BCG, vaccination with BCGΔBCG1419c increased the levels of CD4^+^ and CD8^+^ T lymphocytes, and reduced 1-log_10_ bacterial burden in lungs after 24 weeks post-infection, with reduced pneumonia, indicating its potential as a preventive vaccine against chronic TB (Pedroza-Roldán et al., 2016). Furthermore, in a model resembling reactivation from chronic infection, H37Rv bacillary loads were reduced more than 1-log_10_ after 1 month of corticosteroid treatment, and TB pneumonia was reduced by 30% (Pedroza-Roldán et al., 2016), suggesting that BCGΔBCG1419c could also be considered as a candidate for a post-exposure vaccine. In this regard, immunization with BCG in PPD-positive humans who received a preventive therapy with isoniazid resulted in increased levels of CD3^+^CD56^+^ NKT-like cells (Suliman et al., 2016), suggesting that BCG, a recombinant BCG or other vaccine candidate could be applied to and protect human beings with presumptive or confirmed latent infection.

In this study, C57BL/6 mice were vaccinated with BCG or BCGΔBCG1419c and evaluated for specific immune responses in lungs, spleen, and lymph nodes after 30 days. Mice were also challenged with a low dose aerosol infection with *M. tuberculosis* H37Rv and assessed for specific immune response, cytokine profiles, bacterial burden, and lung lesions after long-term infection (6 months), a condition already tested in BALB/c mice to represent chronic infection (Pedroza-Roldán et al., 2016). Our results indicate that compared to non-vaccinated mice, BCGΔBCG1419c was the only vaccine capable of reducing IL-6, TNF-α, and IL-10 in lungs of infected mice, and significantly reduce perivasculitis, bronchiolitis, and total lung score. We further went on to study transcriptional differences between BCG and BCGΔBCG1419c that might partially explain these findings. This study confirms our previous results and further supports the need for additional studies of BCGΔBCG1419c as a pre- or post-exposure vaccine for chronic TB or reactivation from such state of infection.

## Materials and Methods

### Bacterial Culture for RNA Isolation

Duplicate cultures of BCG wild type and BCGΔBCG1419c in Sauton media were grown as 50 ml cultures in 75 cm^2^ tissue culture flasks, at 37°C, 5% CO_2_, for 10 days. The experiments were repeated, with cells frozen up to completion of a set of three independent experiments, and later, for each 1 mg of wet mass, 1 ml Trizol (Invitrogen) was added, and lysis was performed using 0.4 ml of 0.1 mm zirconia/silica beads by three cycles at full speed in a bead beater, including incubation in ice for 30 s between cycles of beating, followed by 1 min of centrifugation at 13,000 rpm, 4°C. Trizol supernatant was transferred to a heavy-lock phase tube (Eppendorf) containing 200 μl chloroform (Sigma), and vigorously shaken for 15 s, during 2 min. After 10 min at room temperature (RT), we centrifuged at 13,000 rpm, RT, for 5 min, and the supernatant (volume ∼540 μl) was transferred to 1.5 ml tubes, to which 270 μl of 2-propanol (Sigma) and 270 μl of 3 M sodium acetate pH 5.2 (Molecular biology grade, Sigma) were added, followed by mixing and incubation overnight at 4°C. Samples were centrifuged at 13,000 rpm, 4°C, 10 min, and the nucleic acids pellet was washed twice with 70% ethanol. After drying and dissolving in RNAse-free water (Sigma), we quantitated nucleic acids using a UV spectrophotometer. We performed genomic DNA digestion in solution using RQ1 DNase (Promega) following the manufacturer’s recommendation, and further clean-up was performed using RNeasy kit (Qiagen) according to their recommended protocol. RNA was eluted and quantitated, and 4 μg were used for library construction.

### RNASeq and Data Analysis

Quality and purity of RNA samples were determined with a 2100 Bioanalyzer (Agilent, Santa Clara, CA, United States). Illumina RiboZero bacteria kit (Illumina, San Diego, CA, United States) was used for rRNA depletion. TrueSeq Stranded mRNA HT library preparation kit (Illumina, San Diego, CA, United States) was used, to then sequence on the NextSeq sequencing instrument in mid output 150 v2 flow cell. Paired-end 75 bp reads were checked for technical artifacts using FastQC (Andrews, 2010) following Illumina default quality filtering steps. Reads were further trimmed for quality scores and cleaned up for adapter contamination with cutadapt (Martin, 2011). Alignment of reads to reference was performed using STAR (Dobin et al., 2013) with modification of recommended parameters where appropriate. Read counts were collected by using HTSeq (Anders et al., 2015) followed by normalization and analysis with DESeq2 R package (Love et al., 2014). Raw and processed files are stored in Gene Expression Omnibus (GEO) under access number GSE113888.

### Animals

Specific pathogen-free female 6–8 weeks old C57BL/6 mice (The Jackson Lab, Bar Harbor, ME, United States) were maintained at Colorado State University, in the Animal Biosafety Level 3 facility in isolator caging and supplied with sterile and water *ad libitum*. All methods and procedures were performed in accordance with relevant guidelines, and the CSU Institution Animal Care and Use Committee approved all procedures for mouse handling and experimentation prior to commencement of studies (Protocol number: 16-6369A).

### Mycobacterial Strains and Growth Conditions for Vaccination and Infection

*Mycobacterium tuberculosis* H37Rv (TMCC#102) was grown initially as a pellicle on Proskauer and Beck (P&B) medium, then passaged three times in P&B media containing 0.05% Tween 80 to mid-log phase and a working stock maintained at -80°C. *Mycobacterium bovis* BCG Pasteur and its isogenic derivative BCGΔBCG1419 have already been described (Flores-Valdez et al., 2015), and were grown in P&B medium with 0.01% Tween 80 to mid-log phase. Aliquots were stored at -80°C and thawed before use. Mice were vaccinated with 5 × 10^4^ CFU via the subcutaneous route and infected with virulent *M. tuberculosis* H37Rv via the aerosol route using the Glas-Col Aerosol Exposure Chamber (Glas-Col, Terre Haute, IN, United States) using the standard exposure protocol to deliver approximately 100 CFU of bacilli per mouse.

### Bacteriology Assessment of Mycobacteria

The number of colony forming units (CFU) in the lungs and spleens from infected mice were determined at day 180 post-infection. Organs were excised, homogenized in PBS + 0.01% Tween 80 and 10-fold serial dilutions plated onto 7H11 agar plates, which were incubated at 37°C for 14–21 days. The numbers of colonies were counted at the dilution on which 30–300 colonies could be counted. Data were expressed and analyzed as the Log_10_ CFU per organ.

### Determination of T-Cell Activation by IFN-γ ELISpot Analysis

Lung cells were isolated using Liberase^TM^ TM (Roche, Switzerland) and spleen and lymph nodes cells were isolated by mechanical disaggregation as described previously (Grover et al., 2009). For post-vaccination ELISpot, inguinal, axillary, and brachial lymph nodes were used. For post-infection ELISpot, tracheal bronchial, axillary, brachial, and inguinal lymph nodes were employed. Cells (2 × 10^5^) were then cultured in plates coated with anti-IFN-γ capture antibody according to the manufacturer’s protocol (eBiosciences, San Diego, CA, United States) for 24 h at 37°C, 5% CO_2_ in RPMI-1640 supplemented with 10% fetal bovine serum (FBS; Atlas Biologicals, Fort Collins, CO, United States), 100 U/ml penicillin, 100 μg/ml streptomycin, and 200 mM L-glutamine (Sigma, St. Louis, MO, United States) in the presence of *M. tuberculosis* H37Rv-derived culture filtrate protein (BEI Resources, Manassas, VA, United States). Controls included cells incubated in media alone. Spot forming units (SFU) were determined according to the manufacturer’s protocol and the plates were then analyzed by quantifying the number of spots produced by cytokine producing cells using the Series 5 UV-Immunospot Analyzer (C.T.L., Shaker Heights, OH, United States). Analysis of T-cell activation was also performed on lung, spleen, and lymph node cells at day 180 post-infection.

### Post-infection Th1/Th2/Th17 Cytokine Analysis

Lung homogenates from infected mice were analyzed using the Cytometric Bead Array (CBA) assay for Th1/Th2/Th17 cytokines (BD Biosciences, San Jose, CA, United States). The cytokines analyzed included TNF-α, IFN-γ, IL-2, IL-17A, IL-6, IL-4, and IL-10. The assay was performed according to the manufacturer’s protocol and the samples read on a FACSCanto II flow cytometer running FACSDive^TM^ software (BD Biosciences, San Jose, CA, United States) and analyzed using the FCAP Array Software v3.0 (BD Biosciences, San Jose, CA, United States).

### Post-infection Histological Analysis

A lobe of lung from each mouse was inflated and stored in 10% formalin, after which organs were embedded in paraffin, sections cut, and stained with H&E. A qualified pathologist read the slides without prior knowledge of the groupings. A scoring system (0 = no apparent changes to 5 = severe changes) that involved examination of lungs for peribronchiolitis, perivasculitis, alveolitis, “Granuloma” formation, and degree of necrosis was used to give a total lung score for lungs from each mouse. Lesions were assessed as previously described (Taylor et al., 2012). Briefly, the number of lesions apparent in a section was counted and the percentage of involved parenchyma estimated. The following features were assessed individually: peribronchiolitis, perivascular leukocyte infiltration (“perivasculitis”), alveolitis, “granuloma” formation (i.e., granulomatous inflammation), and necrosis on a scale of 0–5 [0 = within normal limits (no change); 1 = minimal changes; 2 = mild changes; 3 = moderate changes; 4 = marked changes; 5 = very severe changes].

### Statistical Analysis

The significance of differences between experimental groups was evaluated by one-way analysis of variance (ANOVA), with pairwise comparison of multi-grouped data sets achieved using Tukey’s *post hoc* test.

## Results

### Mice Vaccination With BCGΔBCG1419c Increased the Levels of IFN-γ Producing Cells in Lungs, Spleen, and Lymph Nodes

**FIGURE 1:**
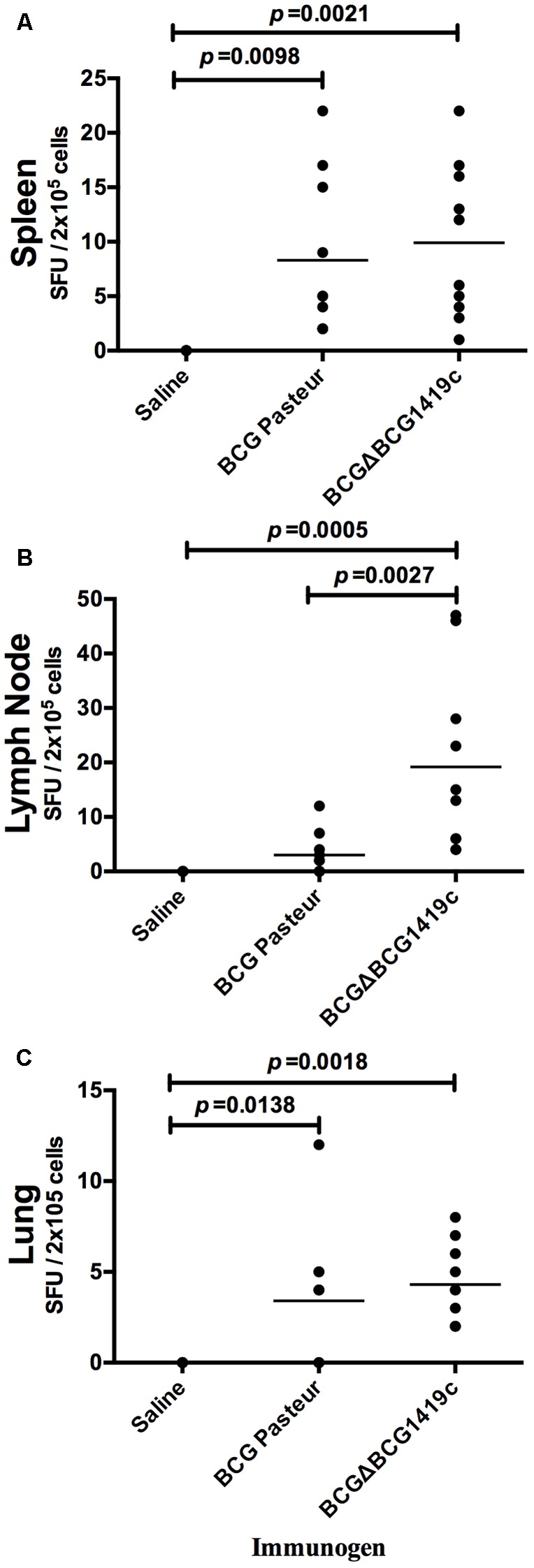
Vaccination with BCGΔBCG149c increases production of IFN-γ in lymph nodes. C57BL/6 mice (*n* = 5) were vaccinated with BCG Pasteur or BCGΔBCG149c (s.c. 5 × 10^4^ CFU), or mock-vaccinated with sterile saline as control. Thirty days after vaccination, cells (2 × 10^5^) obtained from **(A)** spleens, **(B)** lymph nodes, and **(C)** lungs were cultured with culture filtrate antigen (from *M. tuberculosis* H37Rv) for 24 h, and the levels of IFN-γ production were determined. Data (SFUs/2 × 10^5^ cells) from individual samples are shown, with the mean value for each group indicated by a horizontal line. Data were analyzed for statistical significance between the groups by ANOVA, and the resulting *p*-values are shown.

To determine if there were differences between BCG preparations in their ability to activate T cells, single-cell suspensions from the lung, spleen, and lymph nodes of vaccinated mice were analyzed by IFN-γ ELISpot assay. C57BL/6 mice (*n* = 5 per group) were vaccinated as described in the section “Materials and Methods.” After 30 days, animals were euthanized and lungs, spleen, and lymph nodes were removed and processed into single cells suspension. Cells were co-cultured in the presence of culture filtrate antigen derived from *M. tuberculosis* H37Rv and after 24 h, SFUs were determined. As shown in **Figures 1A,C**, the mean SFU values from individual mice were highly variable in BCG-vaccinated groups, still, spleen and lungs of mice administered with BCGΔBCG1419c slightly increased numbers of SFUs in comparison to groups administered with BCG Pasteur. Both groups were statistically significant (*p* < 0.05) in comparison with the saline-administered group. In lymph nodes, vaccination with BCGΔBCG1419c significantly increased (*p* < 0.05) the numbers of IFN-γ-secreting cells, compared with the group vaccinated with BCG and saline (**Figure 1B**). These results show that both BCGΔBCG1419c and BCG activate T lymphocytes equally well in lungs and spleens of vaccinated mice, and that the BCGΔBCG1419c vaccine candidate improved such activation over BCG in lymph nodes. Non-stimulated cells showed mean (SEM) number of spots as follows: Lymph nodes = 1.25 (0.41), lungs = 1.25 (0.95), and spleens = 0.25 (0.25). These values were not shown because they are basically baseline and will not be readily visible in the graph.

### Vaccination of Mice With BCGΔBCG1419c Reduced *M. tuberculosis* Bacterial Load After Low Dose Aerosol Challenge With *M. tuberculosis* H37Rv, Similarly to BCG

**FIGURE 2:**
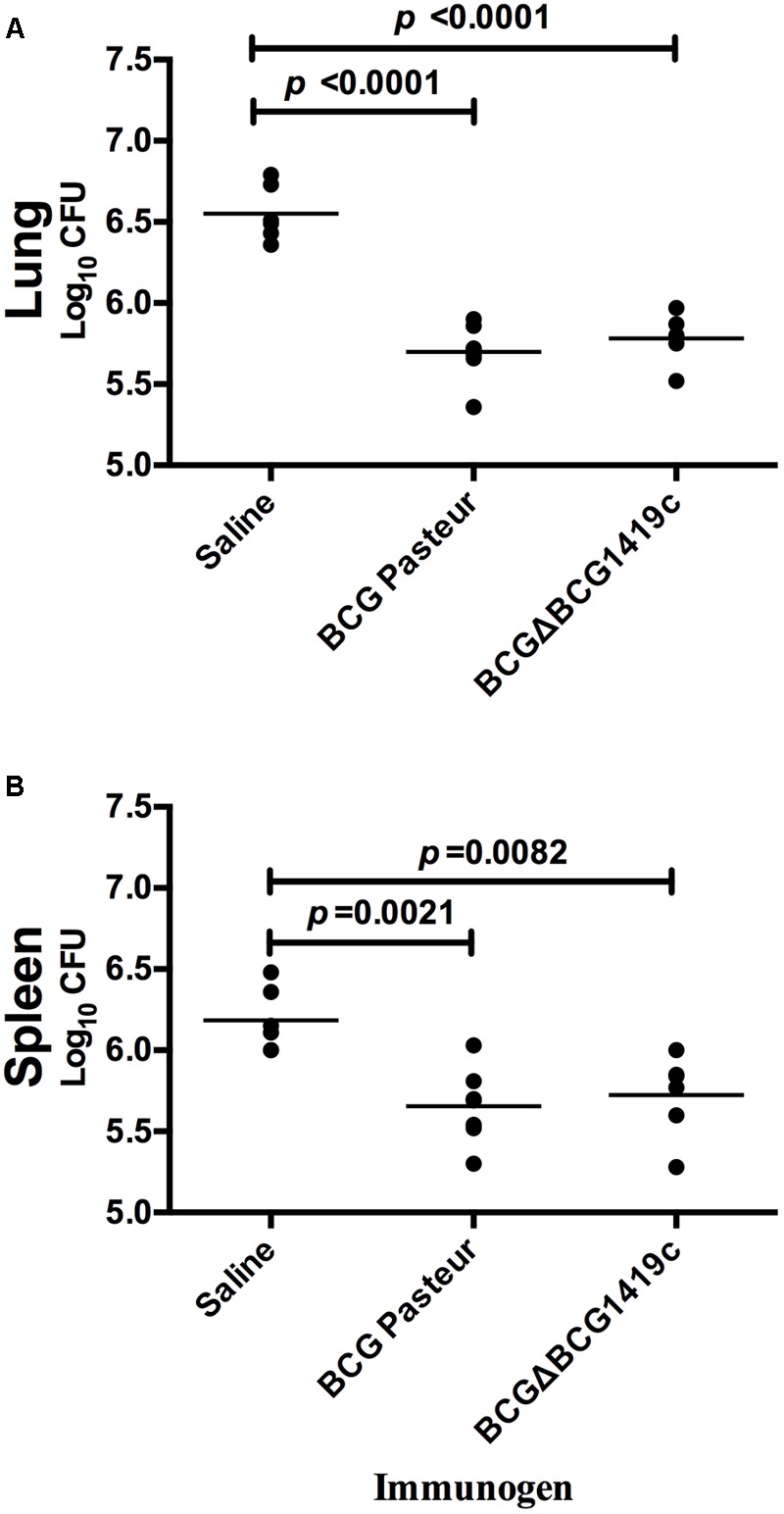
Vaccination with BCG or BCGΔBCG149c provide protection against *M. tuberculosis* replication after aerosol infection. C57BL/6 mice (*n* = 7) were vaccinated with BCG Pasteur or BCGΔBCG149c (s.c. 5 × 10^4^ CFU), or mock-vaccinated with sterile saline as control. Four weeks after vaccination, the mice were challenged with approximately 100 CFU of *M. tuberculosis* H37Rv by aerosol route and the bacterial load was assessed 6 months later in the **(A)** lung and **(B)** spleen. Data (Log_10_ CFU) from individual samples are shown, with the mean value indicated by a line. Data were analyzed for statistical significance between the groups by ANOVA, and the resulting *p*-values are shown.

After 30 days post-vaccination of mice (*n* = 7) with BCGΔBCG1419c, BCG Pasteur, or saline solution, a low dose aerosol challenge with *M. tuberculosis* H37Rv was performed. Then, after 180 days, mice were sacrificed; lungs and spleens were excised and homogenized to determine *M. tuberculosis* bacterial load. As shown in **Figure 2A**, vaccination with BCGΔBCG1419c and unmodified BCG decreased bacterial load (an average of 0.87-log) in lungs of challenged mice, compared with the saline-administered group; however, no statistically significant difference was observed between vaccinated groups. Similarly, spleens obtained from both groups showed an average reduction of 0.91-log compared with the saline-administered group, but with no statistical difference between the BCG-vaccinated groups. Thus, both BCG vaccines tested in this model are equally effective in controlling *M. tuberculosis* H37Rv replication in lungs and spleen after 6 months post-infection.

### Vaccination of Mice With BCGΔBCG1419c or BCG Result in Similar Immune Cell Activation in Response to CFP at 6 Months Post-infection

**FIGURE 3:**
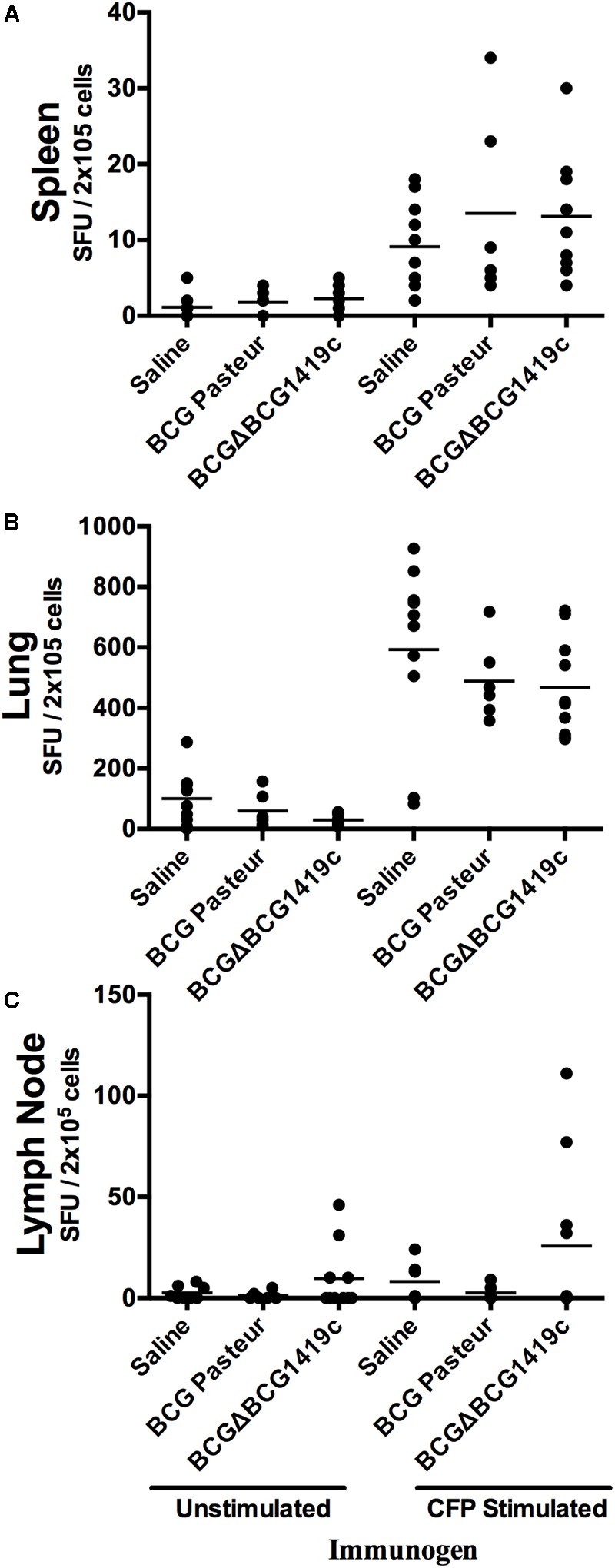
Vaccination with BCG or BCGΔBCG149c does not change immune cells activation in response to CFP after infection. C57BL/6 mice (*n* = 5) were vaccinated with BCG Pasteur or BCGΔBCG149c (s.c. 5 × 10^4^ CFU), or mock-vaccinated with sterile saline as control. Four weeks after vaccination, the mice were challenged with approximately 100 CFU of *M. tuberculosis* H37Rv by aerosol route, and 6 months post-infection, lysates from **(A)** spleen, **(B)** lymph nodes, and **(C)** lungs, and stimulated or not with *M. tuberculosis* H37Rv CFP. Data (SFUs/2 × 10^5^ cells) from individual samples are shown, with the mean value for each group indicated by a horizontal line. Data were analyzed for statistical significance between the groups by ANOVA, and we found variable response from animal to animal, with no statistical significance between vaccinated and non-vaccinated mice.

In order to determine whether immunization with BCG or BCGΔBCG1419c would differentially modulate the immune response after infection, we removed spleen, lung, and lymph nodes of vaccinated mice (*n* = 5) after 180 days post-aerosol challenge. Single cell suspensions were obtained from these organs and co-cultured in the presence of culture filtrated proteins (CFPs) derived from *M. tuberculosis* H37Rv on IFN-γ-coated plates. All groups of mice stimulated with CFP had higher SFU values compared to non-stimulated controls (left vs. right panel, **Figure 3**) with no statistically significant difference among groups stimulated with CFP. The median values of SFU/2 × 10^5^ cells observed in unstimulated vs. CFP-stimulated cells were 2–3 against 10–15 in spleens (**Figure 3A**), 50–100 against 500–600 in lungs (**Figure 3B**), and 15 against 30 in lymph nodes and mostly found in BCGΔBCG1419c-vaccinated mice (**Figure 3C**), respectively.

### Vaccination of Mice With BCGΔBCG1419c Reduces Production of IFN-γ, TNF-α, IL-6, and IL-10 After *M. tuberculosis* Aerosol Challenge

**FIGURE 4:**
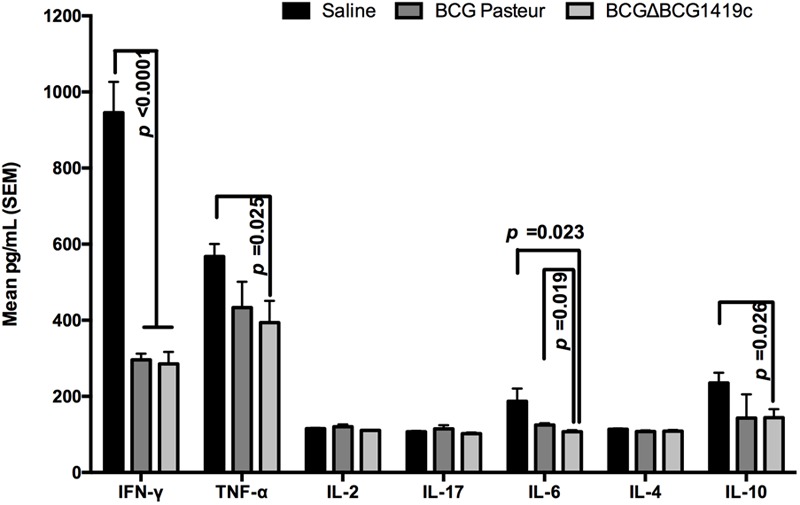
Vaccination with BCGΔBCG149c reduces production of IFN-γ, TNF-α, IL-6, and IL-10 after infection. C57BL/6 mice (*n* = 6–7) were vaccinated with BCG Pasteur or BCGΔBCG149c (s.c. 5 × 10^4^ CFU), or mock-vaccinated with sterile saline as control. Four weeks after vaccination, the mice were challenged with approximately 100 CFU of *M. tuberculosis* H37Rv by aerosol route, and 6 months post-infection, lysates from lungs were used to quantify IFN-γ, TNF-α, IL-2, IL-17, IL-6, IL-4, and IL-10. Data (mean pg/ml ± SEM) were analyzed for statistical significance between the groups by ANOVA, and the resulting *p*-values are shown.

After observing that BCGΔBCG1419c was more effective than parental BCG in inducing immune response in lymph nodes, and equally effective in controlling *M. tuberculosis* H37Rv replication, as well as in inducing T-cell activation during chronic infection, we decided to determine the local immune response produced in the main target of *M. tuberculosis* infection. For this, lungs obtained post-challenge were homogenized for Th1/Th2, and Th17 cytokine production profiling (*n* = 6–7) (**Figure 4**). IFN-γ quantification showed a significant reduction (*p* < 0.001) in IFN-γ concentration in vaccinated groups compared with saline administered. Mice immunized with BCGΔBCG1419c showed significantly lower levels of TNF-α compared with the saline group (*p* < 0.05). IL-2, IL-4, and IL-17 production was similar in the three groups of mice. Production of IL-6 was significantly reduced in mice vaccinated with BCGΔBCG1419c compared with the BCG Pasteur control group (*p* < 0.05) and with the saline group (*p* < 0.05). Finally, a significant reduction (*p* < 0.05) in IL-10 was found in BCGΔBCG1419c-vaccinated mice compared with the saline control group, but not in comparison with BCG Pasteur. These results indicate that BCGΔBCG1419c promoted a diminished inflammatory response at 6 months post-infection, significantly better than parental BCG for IL-6, and also significantly reduced for TNF-α while BCG did not reach significance; moreover, it was equally effective in reducing IFN-γ as unmodified BCG.

### Vaccination With BCGΔBCG1419c Reduces the Levels of Damage Associated With Inflammation in Lungs After *M. tuberculosis* Aerosol Challenge

**FIGURE 5:**
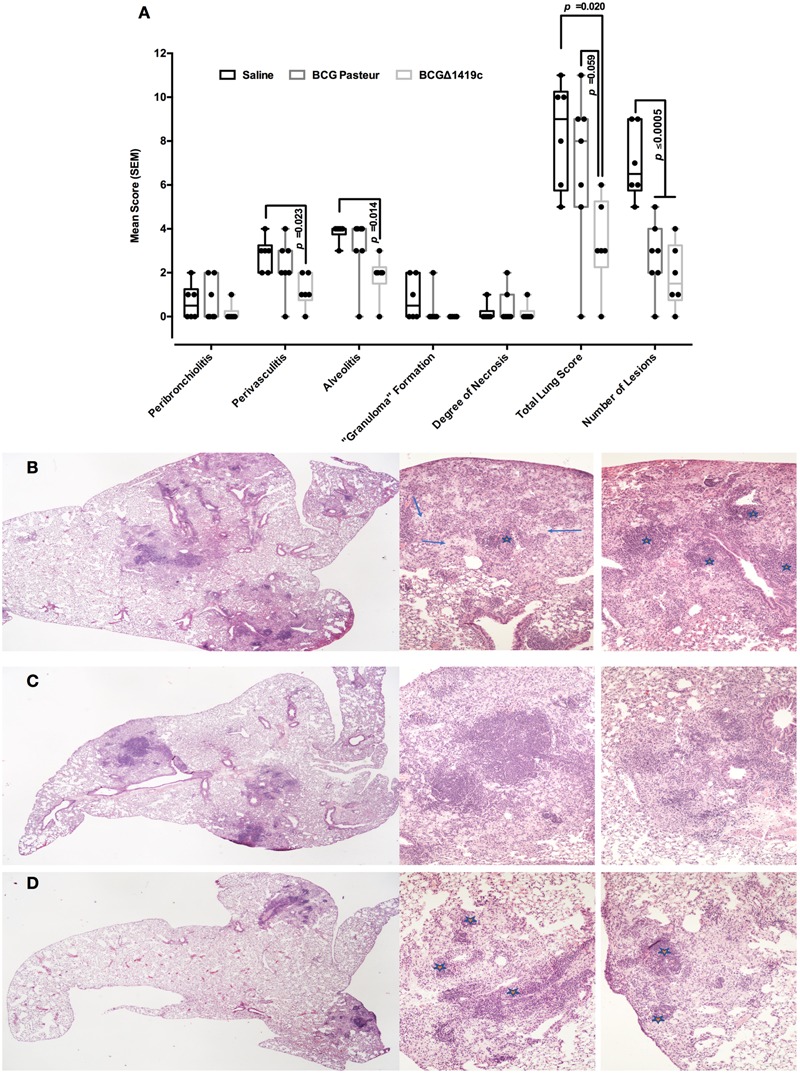
Vaccination with BCGΔBCG149c reduces lung pathology after long-term *M. tuberculosis* aerosol infection. C57BL/6 mice (*n* = 6–7) were vaccinated with BCG Pasteur or BCGΔBCG149c (s.c. 5 × 10^4^ CFU), or mock-vaccinated with sterile saline as control. Four weeks after vaccination, the mice were challenged with approximately 100 CFU of *M. tuberculosis* H37Rv by aerosol route, and 6 months post-infection, lung samples were submitted for blind histological analysis and H&E-stained sections were scored based on the following scoring system: 0 = no apparent changes, 1 = minimal changes, 3 = mild changes, 4 = marked changes, 5 = severe changes. **(A)** Peribronchiolitis, perivasculitis, alveolitis, granuloma formation, degree of necrosis, total lung score, and number of lesions, as assessed with the described scoring system. **(B)** Representative image of animals that received saline, **(C)** BCG Pasteur, or **(D)** BCGΔBCG149c. In **(A)**, data (score) from individual samples are depicted as box and whiskers, showing minimum and maximum values. Data were analyzed for statistical significance between the groups by ANOVA, and the resulting *p*-values are shown. **(B)** Lesions with moderate to severe granulomatous alveolitis and perivasculitis (stars), or with multifocal necrosis (arrows). **(D)** Mild to moderate alveolitis and perivasculitis only (stars).

In order to determine the levels of structural changes associated with inflammation, mice from vaccinated and unvaccinated groups were euthanized at 180 days post-challenge with *M. tuberculosis* H37Rv, and the lungs processed for histopathological assessment. As shown in **Figure 5A**, mice vaccinated with BCGΔ1419c had less tissue damage, particularly and significantly compared to the saline-administered group, when assessed for perivasculitis (*p* = 0.023), alveolitis (*p* = 0.014), total lung score (*p* = 0.020, and *p* = 0.059 vs. BCG-group), and number of lesions (*p* < 0.005). Mice receiving saline showed extensive pneumonia and similar inflammatory infiltrate around blood vessels and airways (average of six lesions per lung in non-vaccinated mice, **Figure 5B**). In comparison, mice vaccinated with BCG Pasteur showed patches of pneumonia and tended to have more severe perivascular and peribronchiolar inflammatory infiltrates (**Figure 5C**), possibly reflecting a vigorous immune response. The BCGΔBCG1419c-vaccinated mice had significantly fewer lesions consisting of minimal to mild alveolitis, accompanied by mild perivascular and peribronchial inflammatory infiltrates of mainly lymphocytes (**Figure 5D**). In fact, the cellular infiltrates were composed mainly of lymphocytes and monocytes in the perivascular and peribronchiolar infiltrates. Alveolar infiltrates were initially mainly macrophages with some lymphocytes in the alveolar septae, but as the lesions take on a more granulomatous appearance, the alveolar infiltrates were also mixtures of macrophages and lymphocytes. Areas with necrosis had infiltration of some/few neutrophils, but they never dominated. The necrosis appeared to affect initially large foamy macrophages in the alveoli and may extend to other cell types if extensive. These findings indicate improved protection versus lung pathology upon vaccination with BCGΔBCG1419c.

### Transcriptional Downregulation in BCGΔBCG1419c in Comparison to BCG Wild Type Might Correlate With Decreased Pathology Upon Immunization

Transcriptomic analysis (RNA-seq) was performed after cultivating BCGΔBCG1419c and BCG Pasteur strains *in vitro*, for 10 days, in Sauton media. As shown in **Table 1**, 32 genes were differentially expressed in BCGΔBCG1419c compared with BCG Pasteur (Benjamini–Hochberg-adjusted *p*-value < 0.01 and greater than 1.5-fold change). These 32 genes are significantly enriched in the TubercuList categories (Lew et al., 2011) “intermediate metabolism and respiration” and “virulence, detoxification and adaptation” (Benjamini–Hochberg-adjusted *p*-value = 0.006 and *p*-value = 0.0007, respectively). Among the genes involved in “intermediate metabolism and respiration,” all eight genes of a single transcriptional unit required for arginine biosynthesis (*argR, argD, argB, argG, argF, argJ, argH*, and *argC*) were downregulated in the BCGΔBCG1419c strain, including the transcriptional regulator *argR*. Other genes with decreased expression and associated with “intermediate metabolism and respiration” included *BCG_0114* (*Rv0081*), *BCG_0116* (*Rv0082*), and *BCG_0018* (*Rv0085*), which are also part of an eight-gene operon, likely regulated by *Rv0081* (*BCG_0114*). Genes associated with “virulence, detoxification, and adaptation” and differentially expressed (repressed) included the chaperones (*groEL2, groES*) and antitoxins (*vapB31* and *vapB17*). There were an additional four genes (*fabD, acpM, kasA*, and *kasB*) that had decreased relative expression in the BCGΔBCG1419c strain. These four genes are consecutive in the genome and are known to be involved in MA synthesis (Bhatt et al., 2007b). Finally, there are only five genes with increased expression in BCGΔBCG1419c compared to BCG, two of which are the probable transcriptional regulators, encoded by *BCG1420* (*Rv1358*) and *BCG1421* (*Rv1359*). In summary, when grown as surface pellicles, a condition used to manufacture BCG vaccine, BCGΔBCG1419c down-regulates expression of key antigenic molecules such as MAs and chaperones. What is the contribution of such molecules for the improved protection against chronic TB remains to be specifically tested.

**Table 1:** List of genes differentially expressed between BCGΔBCG1419c and BCG wild type in 10-days old pellicles.

Category	Gene	Product or Rv homologous gene	Fold change BCGΔBCG1419c vs. BCG wild type
Mycolic acid synthesis	*BCG3487c*	*groEL1*	0.388
	*BCG2262*	*kasA*	0.524
	*BCG2545c*	*fas*	0.540
	*BCG2260*	*fabD*	0.554
	*BCG2261*	*acpM*	0.570
	*BCG2263*	*kasB*	0.610
Arginine synthesis	*BCG1696*	*argR*	0.404
	*BCG1694*	*argD*	0.449
	*BCG1693*	*argB*	0.454
	*BCG1697*	*argG*	0.488
	*BCG1695*	*argF*	0.506
	*BCG1692*	*argJ*	0.516
	*BCG1698*	*argH*	0.516
	*BCG1691*	*argC*	0.649
DosR-regulon	*BCG0114*	*Rv0081*	0.567
	*BCG0116*	*Rv0083*	0.585
	*BCG2049c*	*Rv2030*	0.632
	*BCG2050c*	*hspX*	0.659
Others	*BCG0479*	*groEL2*	0.342
	*BCG3488c*	*groES*	0.470
	*BCG1630c*	*Rv1592c*	0.524
	*BCG1045c*	*Rv0990c*	0.573
	*BCG2265*	*Rv2248*	0.609
	*BCG2547*	*Rv2526*	0.616
	*BCG0118*	*Rv0085*	0.661
	*BCG2267c*	*glpD1*	0.667
	*BCG0798*	*Rv0748*	0.669
	*BCG3904*	*bfrB*	1.665
	*BCG0559c*	*Rv0516c*	1.712
	*BCG1421*	*Rv1359*	1.900
	*BCG1418c*	*Rv1356c*	1.999
	*BCG1420*	*Rv1358*	5.706

*All genes have greater than 1.5-fold change and Benjamini–Hochberg-adjusted *p*-value < 0.01.*

## Discussion

People harboring latent *M. tuberculosis* remains a vast reservoir to continue TB spread. The current BCG vaccine does not protect versus latent infection, nor is effective in reducing lung pathology. Conversely, uncontrolled inflammatory response leads to caseous liquefaction, cavity formation, access to the airways, and continued transmission to new hosts. Recently, we demonstrated that the BCGΔBCG1419c live vaccine candidate improved control of *M. tuberculosis* H37Rv infection in mice after high-dose intratracheal inoculation (Pedroza-Roldán et al., 2016). These results led us to test the BCGΔBCG1419c vaccine candidate as a prophylactic vaccine in a low dose aerosol challenge model, to mimic the route of transmission to the bacilli in humans (Sharpe et al., 2016). Because our working hypothesis is that biofilms resemble aspects of chronic infection, we think the BCGΔBCG1419c vaccine candidate might improve protection compared to BCG, particularly during/at chronic TB infection, or reactivation from it as already demonstrated in BALB/c mice (Pedroza-Roldán et al., 2016), but that such strain could be only as effective (or even less) than conventional BCG during/at early TB infection.

We observed that both BCG- and BCGΔBCG1419c-vaccinated mice increased the levels of IFN-γ secreting cells in spleen, lungs, and lymph nodes in response to culture filtrate proteins, with BCGΔBCG1419c significantly improving IFN-γ production in lymphatic nodes (**Figure 1**). It could be that the BCG strains tested here replicate differently in lymphatic nodes and affect the availability of antigens presented, as BCG has been demonstrated to remain detectable in lymph nodes of vaccinated mice (Derrick et al., 2015), and induce IFN-γ production by innate lymphoid cells (Steigler et al., 2018). Alternatively, differences in intrinsic capacity to present antigens because of the molecular differences occurring between BCG and BCGΔBCG1419c found here (discussed further below) or others yet to be described could also contribute to the observed effect, so experimental evaluation of capacity to present antigens induced by these strains remains to be conducted.

Vaccination with any of the BCG strains tested here promoted no change in pulmonary levels of IL-2, IL-17, and IL-4, while both BCG strains reduced IFN-γ production in lungs of infected mice after 6 months of infection (**Figure 4**), whereas only vaccination with BCGΔBCG1419c significantly reduced TNF-α (*p* = 0.025) and IL-10 production (*p* = 0.026) (**Figure 4**). Moreover, compared to parental BCG, immunization with BCGΔBCG1419c significantly reduced production IL-6 (*p* = 0.019). It has been reported that IL-6 has an inhibitory effect upon adaptive immunity by affecting macrophage response to IFN-γ (Nagabhushanam et al., 2003). On the other hand, treatment of mice with chronic TB (4–6 months post-infection) with an anti-TNF-α antibody resulted in rapid death, with disorganized granulomas and a modest increase in bacterial burden (Mohan et al., 2001), although of course in our work we are not abrogating TNF-α function as opposed to Mohan et al. (2001). Finally, during chronic TB infection of C57BL/6 mice, increased expression of IL-10 resulted in increased bacillary loads in lungs, macrophage accumulation, reduced TNF-α and IL-12p40, and a decreased IFN-γ secretion (Turner et al., 2002). Thus, the lesser inflammation observed at 6 months after challenge with *M. tuberculosis* H37Rv, in mice vaccinated with BCGΔBCG1419c, could be due to an adequately balanced inflammatory response. In this regard, it has been observed that pathological inflammation was promoted via granulocytic inflammation (Mishra et al., 2017), where IL-6 together with IFN-γ and TNF-α could exacerbate extensive tissue damage (Marzo et al., 2014). Based on our findings, it seems likely that BCGΔBCG1419c helps maintaining a good balance in the inflammatory response, which has been suggested as optimal to contain infection and prevent transmission to new hosts (Vyas and Goswami, 2017).

Transcriptomic analysis via RNASeq allowed us to find decreased expression in the BCGΔBCG1419c strain compared to wild-type BCG of several genes (**Table 1**), including *fabD, acpM, kasA*, and *kasB*, which participate in elongation of the meromycolate chain of MAs (Bhatt et al., 2007b). It is known that MA can associate to trehalose to form trehalose dimycolate (TDM, cord factor) [reviewed in Bhatt et al. (2007b)], which is loosely bound to the surface of virulent *M. tuberculosis*. TDM possesses several immunomodulatory properties relevant for TB pathology (Hunter, 2016) and has recently been shown to be more abundantly produced by clinical strains obtained from pulmonary sites compared to extra-pulmonary isolates (Arora et al., 2016).

While *kasA* is essential for *in vitro* replication of *M. tuberculosis* (Sassetti et al., 2003), a *Rhodococcus equi* (an actinomycete related to *M. tuberculosis*) *kasA* mutant is viable and produces 10 carbon units shorter MA compared to wild-type bacteria (Sydor et al., 2013). Coating *Escherichia coli* with shorter-MA-containing TDM did not reduce phagolysosome formation, as opposed to bacteria covered with TDM extracted from wild-type *R. equi* (Sydor et al., 2013). In *M. tuberculosis*, deletion or genetic engineering of *kasB* to emulate a constitutively phosphorylated KasB (inactive) led to increased persistence with reduced lung pathology in immunocompetent mice (Bhatt et al., 2007a; Vilcheze et al., 2014). Moreover, Vilcheze et al. (2014) reported that their *kasB* mutants produced four to six carbon atoms shorter MA chains. Thus, these studies support the notion that other than fine modulation of MA structure by *cis*- or *trans*-cyclopropanation, acyl chains length of MA also impact persistence and lung pathology. We hypothesize that the reduced expression of these genes in BCGΔBCG1419c might allow it to produce MA species that differ from wild-type BCG, and that facilitate its establishing a chronic infection (Flores-Valdez et al., 2015).

We are aware that in a previous report (Flores-Valdez et al., 2015) we did not detect changes in MA between wild-type BCG and BCGΔBCG1419c, while lack of PDIM and changes in PGLs were distinguished. As for MA, it could be that the method we used for harvesting the pellicles sloughed-off these loosely attached molecules from the surface and therefore we found no difference under the conditions we tested (thin-layer chromatography), or that given the level of change detected in transcription (roughly 40–50% decrease compared to wild-type BCG) we need a more sensitive technique to determine if a difference in the end-product exists. The lack of PDIM and the change in PGL length in BCGΔBCG1419c compared to BCG might also play a role in the differential host response and control of TB infection observed after immunization with either vaccine strain and surely deserves to be explored in another study. We aimed first to determine whether or not BCGΔBCG1419c is more effective than BCG versus chronic/LTBI, to later proceed with mechanistic studies of its efficacy. On the other hand, we also found decreased expression of *hspX* and *groEL2* (**Table 1**), which may account for an overall decreased inflammatory response observed in lungs (**Figure 5**).

In a separate study (Segura-Cerda et al., 2018), by means of iTRAQ, we were able to find differences at the protein level for AcpM, a result in agreement with our transcriptomic data (**Table 1**). Furthermore, we also found different levels of antigenic proteins, such as PstS2, HbhA, DnaK, and 35KDa Ag, which were less abundant in BCGΔBCG1419c in comparison with parental BCG (Segura-Cerda et al., 2018). Whether these particular changes contribute to the protection afforded by BCGΔBCG1419c remains to be formally tested, although we favor the notion that the sum of all changes in protein and lipid antigens account for the improved protection observed with this particular vaccine candidate during chronic TB.

The effects of possibly affecting MA production coupled to the lack of PDIM, longer PGLs, and reduced production of PstS2, HbhA, DnaK, Ag35KDa, HspX, and GroEL2, might allow BCGΔBCG1419c to promote a controlled immune response (shown here as reduced TNF-α, IL-6, and IL-10) that leads to the observed decreased pathology after infection, more evidently represented by the total lung score (medians of 8 and 3 for BCG- and BCGΔBCG1419c-vaccinated mice, respectively, *p* = 0.059) and also for reduced scores in perivasculitis and alveolitis that were significant with respect to saline control only in the BCGΔBCG1419c-vaccinated groups (*p* = 0.023 and 0.014, respectively) (**Figure 5**). For all histological parameters evaluated, with the exception of number of lesions found after infection, vaccination with unmodified BCG or no vaccination resulted very similar (**Figure 5A**). Isolation of TDM from both BCG and BCGΔBCG1419c to determine MA chain lengths, as well as testing whether TDM extracted from these strains induces different granulomatous pathology in lung tissue of mice, should help whether or not there is a link between the putative differences in MA production by these two BCG strains in protection versus lung damage after infection.

No vaccine tested thus far in mice is able to attain *M. tuberculosis* sterilization in infected organs. We found that vaccination with BCG or BCGΔBCG1419c provided similar containment of virulent *M. tuberculosis* H37Rv at 6 months post-infection in both lungs and spleens, and better than saline control in C57BL/6 mice (**Figure 2**). Using the same mouse model, it was found that vaccination with a BCG devoid of a methyl transferase gene (BCGΔmmaA4) reduced the bacterial burden of *M. tuberculosis* Erdman strain in lungs after 2- and 3-month post-infection, but only when DDA/TDB adjuvant was used (Derrick et al., 2012). It could be that our BCGΔBCG1419c strain would change protection afforded upon vaccination if supplemented with an adjuvant, although this remains to be verified, and moreover, adding adjuvants to BCG production by manufacturers is not a common practice nowadays.

We think that the increased protective efficacy afforded upon vaccination with BCGΔBCG1419c observed at 6 moths post-infection in BALB/c mice compared to C57BL/6 mice is related to the increase in *M. tuberculosis* CFU numbers after the plateau has been reached due to the high challenge dose used in the former model (Rook et al., 2009), which is absent in the low-dose model used in this study. Such differences have been proposed to resemble conditions likely to happen in developing and developed countries, respectively (Rook et al., 2009). Furthermore, it is known that C57BL/6 and BALB/c mice differ in their H2 genes (H2^b^ and H2^d^, respectively), which have been shown to affect production by spleen and lymph node cells of cytokines evaluated in this work, such as TNF, IL-10, IL-6, and IFN-γ (Matthews et al., 2000), perhaps contributing to the observed differences too.

To date, no fully effective surrogate marker of TB vaccine efficacy exists. In an attempt to compare the efficacy of live vaccine candidates tested for protection against chronic/subclinical TB infection, we decided to compare CFU and lung pathology reduction reported by several authors and ourselves (**Table 2**). It is worth noting that despite evaluating the same event (chronic/subclinical *M. tuberculosis* infection) differences in infectious dose, route of infection, *M. tuberculosis* strain used for challenge, and even the dose of BCG vaccine applied occur, control of *M. tuberculosis* replication in lungs typically approaches 1-log_10_ reduction, an effect achieved with our BCGΔBCG1419c vaccine candidate. Efficacy studies using *M. tuberculosis* clinical isolates and using other models that reproduce additional aspects of chronic/LTBI or reactivation from it might shed additional light on its potential as a vaccine against latent TB. Using different BCG backgrounds might not necessarily provide radically different results, as BCG Pasteur has been shown to promote one of the best reductions in *M. tuberculosis* H37Rv lung loads in BALB/c mice (Zhang et al., 2016). It would be highly desirable to compare vaccine candidates using the same experimental conditions.

**Table 2:** Efficacy of live attenuated vaccine candidates to control *M. tuberculosis* replication and lung pathology in mouse models of chronic TB infection.

Vaccine tested	Mouse strain	Infectious dose/*M. tuberculosis* strain used for challenge/route of infection	Effect as pre-exposure vaccine	Effect as post-exposure vaccine	Reference
BCGΔ*ureC hly*^+^	BALB/c	200 CFU of *M. tuberculosis* Beijing/W delivered via aerosol	Close to 2-log_10_ reduction in CFU in lungs between days 50–200, compared to BCG.		Grode et al., 2005
			No effect reported versus lung pathology.		
BCGΔ*ureC hly*^+^	BALB/c	30 CFU of *M. tuberculosis* H37Rv delivered via aerosol	Close to 1-log_10_ reduction in CFU in lungs at 90 days post-infection compared to BCG, an effect lost at 200 days post-infection.		Grode et al., 2005
			No effect reported versus lung pathology.		
BCGΔ*ureC hly*^+^	BALB/c	100 CFU of *M. tuberculosis* H37Rv delivered via aerosol		0.5-log_10_ reduction compared to parental BCG, after 14 weeks of deprivation of antibiotic treatment in a subclinical TB infection model (100 CFU of *M. tuberculosis* H37Rv followed by antibiotic treatment and deprivation from it).	Gengenbacher et al., 2016
				No effect reported versus lung pathology.	
BCGΔmmaA4	C57BL/6	200 CFU of *M. tuberculosis* Erdman delivered via aerosol	Close to 0.4-log_10_ and 0.1-log_10_ reduction in CFU in lungs at 1 and 4 months post-infection compared to BCG, respectively.	No effect reported/not tested.	Derrick et al., 2012
			No effect reported versus lung pathology.		
BCGΔBCG1419c	BALB/c	2.5 × 10^5^ CFU of *M. tuberculosis* H37Rv delivered via intratracheal	Close to 0.3-log_10_ reduction in CFU in lungs at 6 months post-infection, compared to BCG.		Pedroza-Roldán et al., 2016
			Reduced pneumonia.		
BCGΔBCG1419c	B6D2F1	1000 CFU of *M. tuberculosis* H37Rv delivered via intratracheal		Close to 1-log_10_ reduction in lung CFU after 1 month of immunosuppression with corticosterone (*M. tuberculosis* challenge followed by vaccination and corticosteroid treatment).	Pedroza-Roldán et al., 2016
				Pneumonia reduced by 30% compared to BCG after immunosuppression for 1 month.	
BCGΔBCG1419c	C57BL/6	100 CFU of *M. tuberculosis* H37Rv delivered via aerosol	No difference compared to parental BCG, close to 1-log_10_ reduction with respect to non-vaccinated mice.		This study
			Reduced perivasculitis, bronchiolitis, and total lung score compared to parental BCG.		

Finally, the extensively characterized antigens and subunit vaccine candidates ESAT-6 and Ag85B, have recently been shown to fail in controlling *M. tuberculosis* infection in lungs of mice, because of T-cell exhaustion due to chronic stimulation, and poor antigen expression during persistent infection, respectively (Moguche et al., 2017). This finding along with the lack of success of the most advanced vaccine candidates in clinical trials to improve protection versus TB make a strong argument to look for other potential vaccine candidates exploring alternate hypotheses. Moreover, the potential of following strategies that encompass from discovery to clinical trials has proven promising results (Kaufmann et al., 2017b). While the role of biofilms in TB remains a matter of ongoing research, this work further demonstrates that BCGΔBCG1419c protects against chronic infection by *M. tuberculosis* in diverse mouse models, calling for additional assays as already suggested here, including assays in models than more closely resemble LTBI. As already mentioned, our hypothesis is that BCGΔBCG1419c would be more effective than parental BCG against chronic/latent TB, and not necessarily against active infection. This apparent drawback might translate into an advantage, as administration of an effective vaccine in highly endemic areas, where many adolescents and adults harbor LTBI, will result in the most cost-effective strategy to decrease TB transmission (Knight et al., 2014).

## Author Contributions

MF-V and AI designed the experiments. MF-V, CP-R, EP, RH-P, and AI wrote the main manuscript. MF-V, MA-S, EP, JT, EC, LI, HB-O, and TB performed the experiments. MF-V, CP-R, EP, NB, RH-P, and AI analyzed the data. All authors reviewed and approved the manuscript.

## Conflict of Interest Statement

MF-V, RH-P, CP-R, and MA-S have filed for patents related to BCGΔ1419c as a vaccine candidate strain against TB. The remaining authors declare that the research was conducted in the absence of any commercial or financial relationships that could be construed as a potential conflict of interest.
